# Antiplatelet Effects of a Combination of Sappan Wood (*Caesalpinia sappan* L.) and Red Ginger (*Zingiber officinale var. Rubrum*) Extracts in a High-Fat Diet-Induced Rat Model

**DOI:** 10.1155/adpp/5543717

**Published:** 2024-12-20

**Authors:** Meidi Utami Puteri, Nur Afifah, Anisa Qisti Mathriul, Farhan Mahmudi Wicaksono, Mellynia Tri Sugiarti, Raihana Izzatinisa, Mitsuyasu Kato, Fadlina Chany Saputri

**Affiliations:** ^1^Laboratory of Pharmacology-Toxicology, Faculty of Pharmacy, Universitas Indonesia, UI Depok Campus, Depok 16424, West Java, Indonesia; ^2^National Metabolomics Collaborative Research Center, Faculty of Pharmacy, Universitas Indonesia, UI Depok Campus, Depok 16424, West Java, Indonesia; ^3^Department of Experimental Pathology, Graduate School of Comprehensive Human Sciences and Faculty of Medicine, University of Tsukuba, 1-1-1 Tennodai, Tsukuba 305-8575, Ibaraki, Japan

**Keywords:** antiplatelet, HFD-induced rat, PCSK9, P-selectin, RANTES, red ginger, sappan wood

## Abstract

**Background:** Antithrombotic medications, including antiplatelet agents, are standard treatments for patients with hyperlipidemia who have a high risk of developing cardiovascular disease (CVD). The ongoing exploration of new antiplatelet agents with minimal bleeding effects is crucial, including the investigation of potential compounds derived from natural products. This study intended to evaluate the antiplatelet effects of a combined extract of sappan wood (*Caesalpinia sappan* L.) and red ginger (*Zingiber officinale* var. *Rubrum*) in high-fat diet-(HFD)-induced rats.

**Methods:** Eighteen male Wistar rats were grouped into six groups (*n* = 3): control, negative, positive, and three groups of various combinations of extracts. All groups, excluding the control group, were fed an HFD for 8 weeks. In the eighth week, the control and negative groups were given carboxyl methyl cellulose (CMC) 0.5%, the positive control group was administered aspirin, and the other three groups were administered the combination extract of sappan wood and red ginger at various doses for 2 weeks. Blood samples were collected to assess the levels of hyperlipidemia and platelet hyperactivity markers by enzyme-linked immunosorbent assay (ELISA). The physiological effects of platelet hyperactivity were evaluated using the tail bleeding assay.

**Results:** HFD-induced hypertriglyceridemia and hypercholesterolemia synergistically enhanced platelet hyperactivity after 8 weeks of induction. Interestingly, administration of all doses of the combined extract for 2 weeks significantly decreased the platelet activation markers P-selectin, RANTES, and PCSK9 in a dose-dependent manner compared with the negative control. In addition, the combination of sappan wood and red ginger extract at dose 3 (sappan wood:red ginger: 200:800 mg/200 bw/day) significantly extended the bleeding time of rats (*p* < 0.05) compared to the negative control.

**Conclusion:** Collectively, our results highlight the antiplatelet effect of a combination of sappan wood and red ginger extract in HFD-fed rats.

## 1. Introduction

Cardiovascular diseases (CVD) are the leading cause of death worldwide, with ischemic heart disease being the most prevalent, as reported in recent data from the Global Burden of Disease [1]. Ischemic heart disease can progress to the point where blood flow to the heart muscle is fully obstructed, leading to a heart attack or myocardial infarction (MI) [2, 3]. The pathophysiology of MI involves the rupture of atherosclerotic plaques, which is frequently cited as the main cause of aggravated atherosclerosis [3, 4]. This process involves platelet activation and the coagulation cascade [3, 4]. Platelet activation also contributes to plaque formation, which can lead to thrombotic occlusion of coronary vessels [3, 4]. Platelet activation plays a role in the initiation, progression, and aggravation of atherosclerosis, thereby increasing the risk of MI [4–6].

Various models have been developed to study atherosclerosis-induced CVD pathogenesis and interventions, including high-fat diet (HFD) animal models [7, 8]. Hyperlipidemia is defined as a lipid metabolism disorder characterized by increased levels of triglycerides and cholesterol, which are major risk factors for thrombotic events and contribute to the development of atherosclerosis-induced CVD [9]. In hyperlipidemia, the development of thrombotic events involves platelet hyperactivation, suggesting that hyperlipidemia triggers excessive platelet activation and exacerbates atherothrombosis [10–12]. Activated platelets release a variety of proteins, including thromboxane A2 (TxA2), P-selectin, RANTES, and platelet factor 4 (PF-4) [13–17]. Recent studies have reported that proprotein convertase subtilisin kexin 9 (PCSK9) is a potential biomarker of CVD and is associated with platelet activation [18–20]. In our previous study, we confirmed that hypercholesterolemia and hypertriglyceridemia were observed in male Wistar rats induced with HFD, resulting in increased platelet activation [21]. This enhancement was indicated by elevated levels of P-selectin, PF-4, and PCSK9 following HFD induction in rats [21].

Due to the crucial role of platelet activation in the advancement of atherosclerosis-induced CVD, individuals diagnosed with hyperlipidemia and at high risk of cardiovascular incidents often receive treatment with antithrombotic medications, including antiplatelet agents, to slow disease progression [6, 22]. However, concerns regarding the bleeding risks associated with current antiplatelet agents highlight the need for ongoing research on new antiplatelet agents that offer minimal bleeding side effects [6, 22]. This has led to the exploration of compounds derived from natural products. Recently, multiple preclinical and clinical studies have investigated the benefits of phytochemical compounds from medicinal plants for their cardiovascular health benefits [23, 24].

Sappan wood (*Caesalpinia sappan* L.) and red ginger (*Zingiber officinale* var. *Rubrum*) are two traditional medicinal plants commonly used in Indonesia and known to contain phenolic compounds [25, 26]. Sappan wood exerts cardioprotective effects [26]. Several metabolites including brazilin, brazilein, and sappanone have been isolated and identified from sappan wood extract [26–28]. Brazilin from sappan wood itself exhibits antithrombotic activity, but additional investigations are needed to clarify its antithrombotic mechanism [24, 29]. In addition to its anti-inflammatory and antioxidant effects, ginger (*Zingiber officinale*) has been recognized for its antiplatelet effects, which are attributed to its gingerol and shogaol contents [30–32]. Interestingly, red ginger reportedly contain high concentrations of (6)-gingerol, (6)-shogaol, and gingerdione compounds that are believed to contribute to its pharmacological effects; however, its antiplatelet effects have not been evaluated [25].

The current study intended to explore the antiplatelet effect of a combined extract of sappan wood and red ginger, considering the enhancement in its therapeutic potential when both extract function synergistically [33–35]. Moreover, these plant combinations have been used in Wedang Uwuh, a traditional ethnic drink from Java, Indonesia, and are known to provide numerous health benefits [36]. Using an established animal model, we investigated potential pharmacological interventions for atherosclerosis-induced CVD by assessing the impact of the combined extract on platelet hyperactivity in HFD-fed male Wistar rats.

## 2. Materials and Methods

### 2.1. Animals

Three-months-old male Wistar rats weighing between 200 and 300 g was used. Animals were purchased from the Faculty of Medicine, Padjajaran University (Laboratory of Pharmacology and Therapeutic), Bandung, Indonesia. The animals were acclimatized for 14 d at the Animal House Center, Faculty of Health Science (RIK) Building, Universitas Indonesia, before initiating the experiments. The conditions were maintained at room temperature (25°C) with a 12-h light/dark cycle. The study adhered to the Animal Act of 1986 and was approved by the Ethics Committee of the Faculty of Medicine, Universitas Indonesia (reference number: KET-213/UN2.F1/ETIK/PPM.00.02/2022).

### 2.2. Chemicals and Extracts

Aspirin, the positive control used in this study, was obtained from PT Medifarma Indonesia (Depok, Indonesia) under the brand name aspilets. Ketamine used as an anesthetic agent was Ket-A-100 (SENASA, Peru). Heparin was purchased from Inviclot (Indonesia). Dried extracts of sappan wood and red ginger were obtained from PT Phytochemindo Reksa (Bogor, Indonesia; Supporting Figures 1 and 2). These extracts were administered to the animals in a suspension dosage form prepared with 0.5% carboxyl methyl cellulose (CMC) at doses that had been previously optimized in a preliminary study [35]. It was orally administered at a dose of 3 mL per 200 g body weight per day for 2 weeks. The variation in extract doses is shown in Table 1.

### 2.3. Experimental Design

The rats were grouped into six groups, each containing three rats (*n* = 3), as outlined in the following (Table 2).

**Figure 1:**
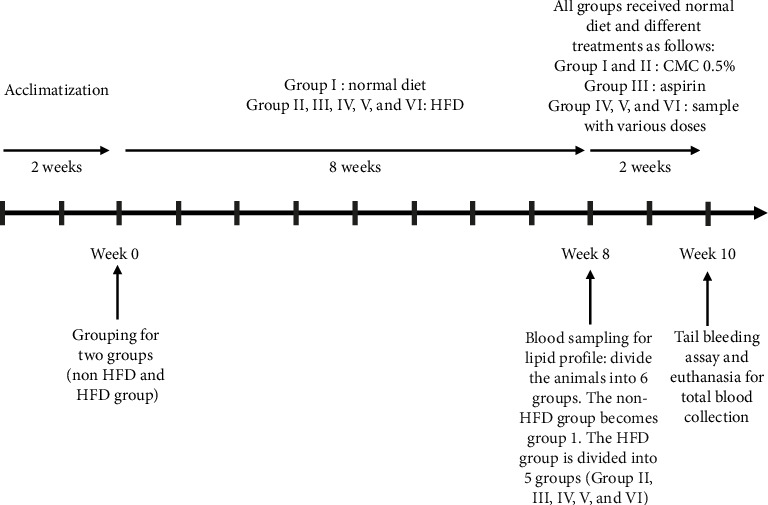
Experimental design of a 10-week study using male Wistar rats.

The experimental design showing the chronological sequence of dietary interventions and treatments administered to different groups is illustrated in Figure 1.

### 2.4. HFD

The treatment groups were fed a HFD daily, as described in a previous study (Table 3) [21]. The HFD was administered orally (p.o.) at a dose of 3 mL/200 g bw/day for 8 weeks.

### 2.5. Blood Collection

Before blood sampling, the rats were fasted for 10–12 h. During weeks 0, 8, and 10, fasting commenced following HFD administration at approximately 8 a.m., and subsequent blood collection occurred 10–12 h later. Blood collection was done typically between 6 and 8 p.m. on the same day. Blood samples were obtained from the sinus orbital at weeks 0 and 8 to assess lipid profiles, while blood collected at week 10 was drawn to assess both lipid profiles and platelet activation markers. In the 10th week of the induction period, all rats were anesthetized with ketamine at a dose of 100 mg/kg body weight by intraperitoneal (i.p.) injection. Blood samples were obtained from the abdominal aorta, with blood volumes ranging from approximately 5–8 mL. The procedure followed a previously described method with some modifications [37]. Blood sampling was carefully performed to avoid lysis. Blood containing heparin was subsequently centrifuged for 10 min at three thousands rpm and the plasma was kept at −20°C for the next step of analysis.

### 2.6. Total Plasma Triglyceride and Cholesterol Assay

Commercially available kits for total triglycerides (Human, Germany) and total cholesterol (Human, Germany) were used to evaluate total plasma triglyceride and cholesterol levels at weeks 0, 8, and 10 in accordance with the manufacturer's guidelines. An absorbance measurement of 500 nm was used to obtain the absorbance value, which was then converted to mg/dL to obtain the total plasma concentration.

### 2.7. Platelet Activation Markers' Measurement

Platelet activation parameters are determined by the plasma levels of platelet hyperactivity biomarkers. Samples were analyzed using commercially available enzyme-linked immunosorbent assay (ELISA) commercially available kits in accordance with the manufacturer's guidelines. All platelet activation biomarkers were measured using rat ELISA kits for PCSK9, PF4, and TxA2 purchased from Wuhan Fine Biotech Co. Ltd. (FineTest ELISA kit, Wuhan, China). Then, samples were analyzed using the Glomax (Multi-detection sytem, Promega, Madison, WI, USA), which measured a wavelength of 450 nm.

### 2.8. Bleeding Time Test

Antithrombotic activity was assessed using a bleeding time assay as previously described [35]. The bleeding time test was conducted one day before the animals were sacrificed for blood collection via the abdominal aorta at week 10. In summary, the rats were placed in a horizontal position and anesthetized with a ketamine dose of 100 mg/kg body weight, if needed. A 20-mm part of the tail was cut from each animal using a scalpel. The injured tail end was placed in a 15 mL Falcon tube containing normal saline buffer. The rest of the tail was positioned vertically, with the tip positioned horizontally approximately 20 mm below the body. Bleeding observations were conducted every 20 min, and differences in bleeding times among the various groups were documented.

### 2.9. Statistical Analysis

GraphPad Prism 10 was used to analyze data statistically. Data are presented as the mean ± standard deviation (SD) of three rats. Differences were assessed using ANOVA followed by a post hoc test, with *p* < 0.05 considered statistically significant.

## 3. Results and Discussion

### 3.1. HFD Induces Hypercholesterolemia, Hypertriglyceridemia, and Body Weight Gain in Wistar Rats

**Figure 2:**
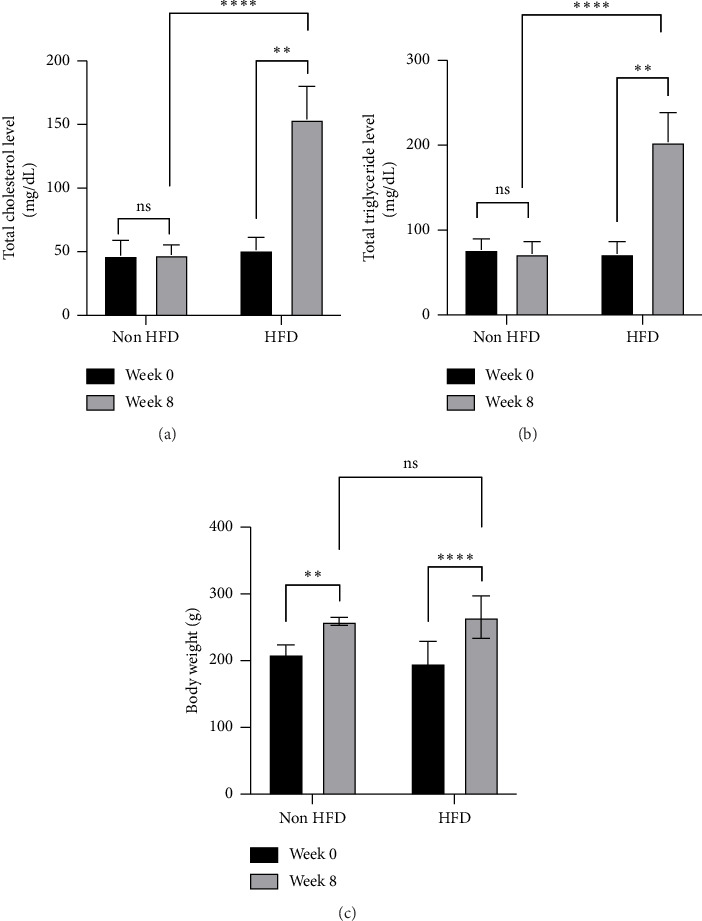
The effect of high-fed diet (HFD) for 8 weeks on (a) total cholesterol, (b) total triglyceride, and (c) body weight in male Wistar rats. Data are presented as the mean ± SD (*n* = 3 rats/group), and ⁣^∗∗^*p* < 0.01 and ⁣^∗∗∗∗^*p* < 0.0001 denote statistical significance.

Our previous study demonstrated that HFD induction in Wistar rats for 8 weeks resulted in hypercholesterolemia and hypertriglyceridemia, with plasma levels equal to or exceeding 140 mg/dL and 120 mg/dL, respectively [21]. In the current study, to confirm that the negative, positive, and treatment groups satisfied hyperlipidemic conditions before administration of the combined extract, we first validated the induction method by dividing the animals into two groups: non-HFD (control) and HFD (negative, positive, and treatment). The HFD group was orally administered HFD daily for 8 weeks, while the non-HFD (control) group received only the aquadest. Blood samples were taken, and plasma cholesterol and triglyceride levels were evaluated. As anticipated, HFD induction in all groups led to increased plasma cholesterol and triglyceride levels after 8 weeks compared with the levels before HFD administration and those in the control group (Figures 2(a) and 2(b)). Hyperlipidemia is a significant risk factor for atherosclerosis-induced CVD [9]. Several animal studies have established an HFD model for mimicking hyperlipidemic conditions, including hypercholesterolemia and hypertriglyceridemia, and have used it as a CVD study model [7, 8]. The consumption of high cholesterol, fructose, saturated fat, and carbohydrates, which contain high calories, causes an increase in total cholesterol, triglycerides, and LDL [7]. Our results indicated that various nutrients, including fat, cholesterol, carbohydrates, and protein, in our HFD used in the study promoted high cholesterol and triglyceride levels. In addition, we observed the effects of the HFD on body weight. An HFD can lead to increased body weight through the accumulation of visceral fat, with the extent of weight gain varying according to the level of fat intake [38]. The present study demonstrated a significant increase in body weight after induction in the HFD group (Figure 2(c)). However, there was no significant difference in the body weight between the HFD and non-HFD groups at week eight (Figure 2(c)).

### 3.2. Combination of Sappan Wood and Red Ginger Extracts Reduces Total Plasma Cholesterol and Triglyceride Levels

**Figure 3:**
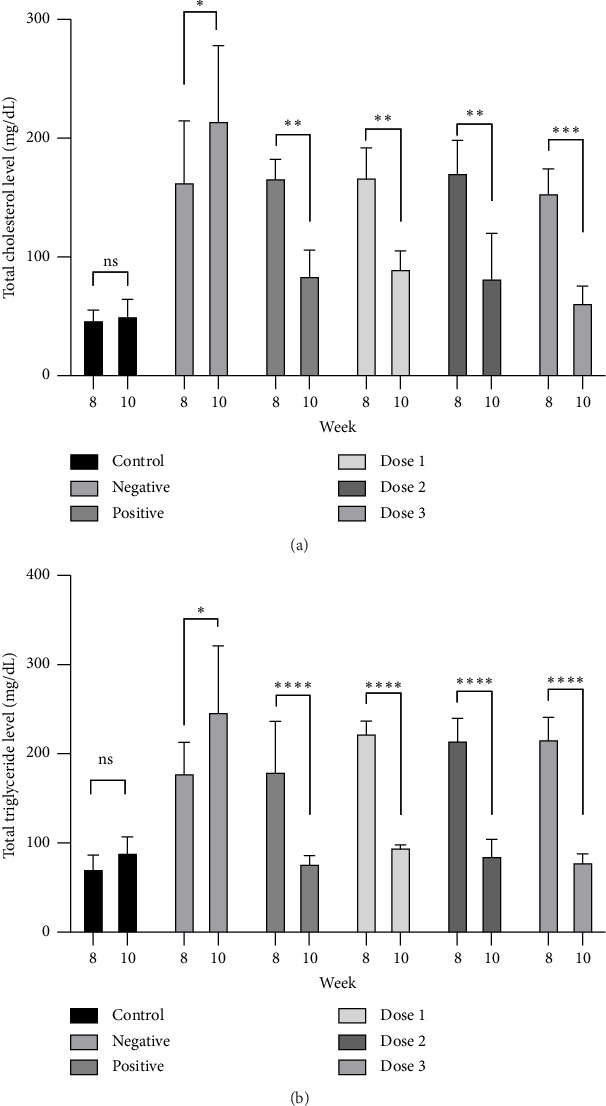
The effect of administration of either CMC (control and negative), aspirin (positive), or various doses of combined extracts (Dose 1, Dose 2, and Dose 3) on (a) total cholesterol and (b) total triglyceride levels 2 weeks after an 8-week HFD induction period in male Wistar rats. Data are presented as the mean ± SD (*n* = 3 rats/group), ns: not significant, and ⁣^∗^*p* < 0.05, ⁣^∗∗^*p* < 0.01, ⁣^∗∗∗^*p* < 0.001, and ⁣^∗∗∗∗^*p* < 0.0001 denote statistical significance.

After all HFD groups developed hypercholesterolemia and hypertriglyceridemia by week eight and were administered either CMC 0.5% (negative), aspirin (positive), or various doses of the combined extract (doses 1, 2, and 3) for 2 weeks, all rats were euthanized to collect blood plasma at week 10. This was performed to evaluate the effect of the combined extract on the lipid profile of HFD-fed rats. Body weight, plasma cholesterol, and triglyceride levels were analyzed. Notably, while we observed no significant difference in body weight change from week 8 to week 10 across all groups (Supporting Figure 3), there was a significant decrease in total plasma cholesterol and triglyceride levels at week 10 in the positive group and the combined extracts at various doses (Figures 3(a) and 3(b)). The positive group used in this study, aspirin, exhibited a significant decrease in plasma levels of cholesterol and triglycerides, consistent with previous studies in which aspirin improved hyperlipidemic conditions in rodent models [39, 40]. Karam et al. reported that after 5 weeks of aspirin administration, total plasma cholesterol, triglycerides, and LDL were significantly decreased in HFD-fed rats compared with the vehicle-treated group [39]. Diepen et al. demonstrated that aspirin, a nonsteroidal anti-inflammatory drug that inhibits the enzyme cyclooxygenase, reduces NF-*κ*B activity. This, in turn, decreased the elevated triglyceride levels induced by HFD in mice by lowering very-low-density lipoprotein (VLDL)-triglyceride secretion from the liver [40]. Interestingly, rats in dose groups 1, 2, and 3 exhibited a significant decrease in both plasma levels of cholesterol and triglycerides compared with those before the administration of the combined extract (*p* < 0.05). These results demonstrate the antihyperlipidemic effects of both extracts, which are consistent with previous studies. A study by Nirvana et al. reported that red ginger can lower triglyceride levels in Wistar rats fed a HFD [25, 41]. The decrease in triglyceride levels was likely due to the presence of niacin in red ginger. Many studies have suggested that niacin leads to an increased clearance of VLDL, resulting in reduced triglyceride levels. In addition, niacin enhances LDL uptake by the liver and inhibits cholesterol synthesis, contributing to an overall reduction in triglycerides observed [42]. Moreover, the reduction in triglycerides and cholesterol by sappan wood extract was consistent with a study by Diana et al., in which the administration of sappan wood extract for 14 days lowered lipid profiles, including triglycerides and cholesterol, in diabetes-induced rats, although the mechanism has not been clearly elucidated [43]. The negative group consistently showed elevated levels of both total cholesterol and triglycerides, as shown in Figures 3(a) and 3(b). In contrast, the control group, which remained free from HFD exposure and received only CMC for 2 weeks, exhibited no significant alterations in cholesterol and triglyceride levels. Collectively, these findings highlighted the effects of aspirin and the combined extract on the lipid profiles of HFD-fed rats.

### 3.3. HFD Induces Platelet Activation, and Aspirin Treatment Reduces Platelet Activation Markers

**Figure 4:**
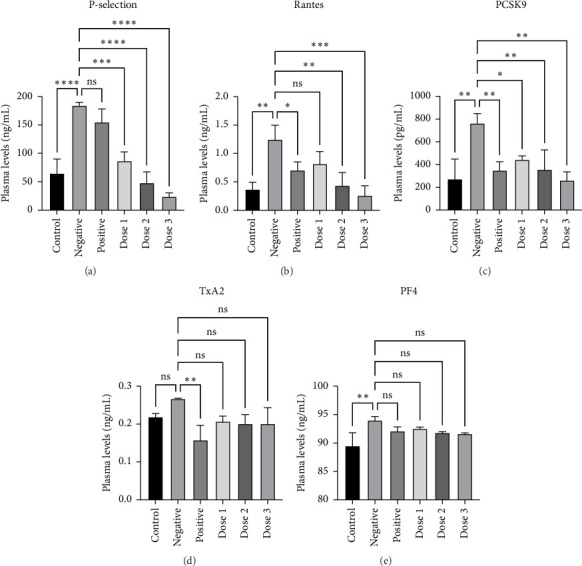
The effect of administration of either CMC (control and negative), aspirin (positive), or various doses of combined extracts (Dose 1, Dose 2, and Dose 3) on plasma levels of platelet activation parameters: (a) P-selectin, (b) Rantes, (c) PCSK9, (d) TxA2, and (e) PF4 for 2 weeks after an 8-week HFD induction period in male Wistar rats. Data are presented as the mean ± SD (*n* = 3 rats/group), ns: not significant, and ⁣^∗^*p* < 0.05, ⁣^∗∗^*p* < 0.01, ⁣^∗∗∗^*p* < 0.001, and ⁣^∗∗∗∗^*p* < 0.0001 denote statistical significance.

After assessed the lipid profile, next, we utilized the blood plasma collected at week 10 to investigate platelet hyperreactivity. Previous studies have suggested an increase in platelet activation in HFD-induced rat models [44]. To better understand this connection and assess the antiplatelet effect of the combined extract, we analyzed the plasma levels of RANTES, P-selectin, PF-4, TxA2, and PCSK9 using ELISA in the tenth week. Our findings indicated that the negative group exhibited higher levels of platelet activation markers, RANTES, P-selectin, PF-4, and PCSK9, but not TxA2, compared to the control group, which was not induced by the HFD (Figures 4(a), 4(b), 4(c), 4(d), and 4(e)). These results are in line with our previous findings, which showed that hypercholesterolemia and hypertriglyceridemia exert a stimulatory effect on several biomarkers of platelet activation, including PF-4, P-selectin, beta-thromboglobulin, and PCSK9, but not TxA2 [21]. The reason why TxA2 is an outlier compared with other platelet activation biomarkers used in this study is still unknown; however, investigations into which plasma cholesterol and triglycerides are associated with increased platelet activation markers have been comprehensively discussed in previous studies [44, 45]. Our data revealed that the positive group receiving aspirin showed a trend toward reduced levels of various platelet markers, including TxA2, PCSK9, RANTES, P-selectin, and PF-4. However, the reduction in PF-4 and P-selectin levels was not statistically significant compared with the negative group (Figures 4(a), 4(b), 4(c), 4(d), and 4(e)). Aspirin is a well-known antiplatelet agent, which functions by inhibiting the activity of cyclooxygenase-1 (COX-1), subsequently blocking the formation of TxA2 in platelets and hindering platelet aggregation [39, 40, 46]. However, the exact mechanism by which aspirin affects platelets beyond the COX-1 blockade remains unclear. One study suggested that aspirin inhibits platelet activation by attenuating reactive oxygen species (ROS) signaling [20]. McKenzie et al. explored the effects of aspirin on the expression of nine platelet surface receptors in whole blood using flow cytometry. Their findings indicated dose-dependent inhibition of P-selectin, CD63, GPIIb/IIIa, and CD107a platelet receptor expression in whole-blood samples treated with aspirin [46]. While studies on the impact of aspirin on the plasma levels of RANTES, PF-4, and PCSK9 in humans are limited, Qi et al. reported that aspirin could cancel the increased-PCSK9 level on platelet activation and thrombosis events in vivo [20].

### 3.4. Combination of Sappan Wood and Red Ginger Extracts Exhibit Antiplatelet Effect by Reducing Platelet Activation Markers P-Selectin, RANTES, and PCSK9

Next, our results demonstrated that the administration of the combined extract at various doses led to a dose-dependent reduction in the plasma levels of P-selectin, RANTES, and PCSK9 (Figures 4(a), 4(b), and 4(c)) compared with the negative group. In addition, we observed a reduction in the TxA2 and PF4 levels, although these reductions were not statistically significant (Figures 4(d) and 4(e)). The link between platelet activation, atherosclerosis progression, and pathogenesis of cardiovascular events is well-documented [3–5]. Interestingly, several studies have highlighted the cardioprotective effects of red ginger and sappan wood owing to their antiplatelet properties [29, 47–49]. Tjendraputra et al. reported that ginger constituents, particularly gingerol and shogaol, which are also abundant in red ginger, exhibit antiplatelet activity in vitro by inhibiting COX-1, a key regulator of TxA2 [47]. Similarly, brazilin, a phytochemical from sappan wood, demonstrated inhibitory effects on ADP- and collagen-induced platelet aggregation both ex vivo and in vivo [29, 48, 49]. Despite the limited research on the direct effects of these extracts on specific antiplatelet markers, the PI3K/Akt signaling pathway is recognized as a key regulator of platelet activation, aggregation, and thrombus formation through the modulation of markers such as P-selectin, integrin, and GPIIb/IIIa [50, 51]. Studies suggest that both red ginger and sappan wood may influence the PI3K/Akt signaling pathway, potentially affecting platelet function and the associated cardiovascular processes [26, 52]. This suggests a plausible mechanism for the antiplatelet effects of the combined extract.

Furthermore, PCSK9 has gained attention for its pleiotropic effects beyond lipid metabolism, particularly for its role in promoting atherothrombosis through enhanced platelet activation and inflammation, which contributes to the progression of atherosclerosis-induced CVD [19, 53, 54]. Qi et al. demonstrated that PCSK9 induces platelet activation by binding to platelet CD36, thereby activating downstream signaling pathways and promoting cardiovascular events, such as MI [20]. Our study found that the combined extract decreased PCSK9 levels, suggesting a potential mechanism of action by which the phytoconstituents from the extract exert their effects by inhibiting PCSK9. Notably, our previous in silico study demonstrated the binding affinity between PCSK9 and brazilin, indicating potential inhibitory functions of brazilin against PCSK9 that may interfere with its activity [55].

**Figure 5:**
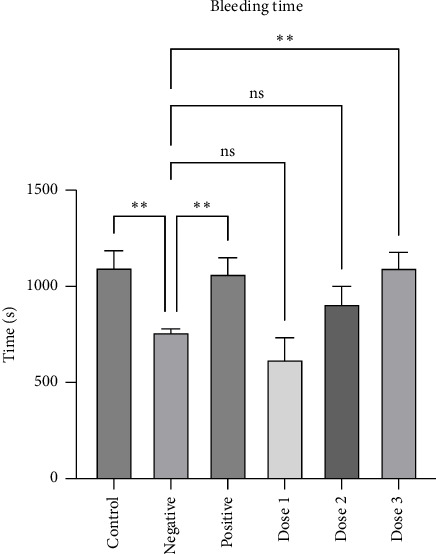
The effect of administration of either CMC (control and negative), aspirin (positive), or various doses of combined extracts (Dose 1, Dose 2, and Dose 3) on bleeding time 2 weeks after an 8-week HFD induction period in male Wistar rats. Data are presented as the mean ± SD (*n* = 3 rats/group), ns: not significant, and ⁣^∗∗^*p* < 0.01 denote statistical significance.

Next, to further assess platelet hyperreactivity, we measured the bleeding time as a physiological parameter and calculated the average bleeding time (Figure 5). Noticeable differences (*p* < 0.05) were noted between the control and negative groups, as well as between the negative and positive groups. Interestingly, both control and positive groups exhibited longer bleeding times than the negative group (Figure 5). These data indicate that HFD induction, which is associated with platelet hyper-reactivity, may prolong bleeding time [11, 12]. Furthermore, our results demonstrated a dose-dependent effect, in which the combined extract prolonged bleeding time compared with the negative group. Specifically, dose 3 (sappan wood:red ginger; 200:800 mg/200 bw) significantly prolonged bleeding time in rats compared to the negative group (*p* ≤ 0.05). The current study is in line with our previous investigation, which reported that the combination of sappan wood and ginger extract has the potential to be an antithrombotic drug, as evidenced by the increased survival rate and bleeding time of the test mice [35]. Taken together, the ELISA results, along with the bleeding time assay, indicated the antiplatelet effect of the combination of sappan wood and red ginger extract, as demonstrated by the inhibition of platelet activation biomarkers and the extension of bleeding time. Furthermore, our study has added additional findings on how combined extracts can enhance pharmacological activity in cardiovascular protection [56, 57]. One of example is study that reported the endothelial protective effects of a combination of *P. baumii* (500 mg/kg) and *S. miltiorrhiza* (100 mg/kg) in a rat model with collagen-epinephrine-induced platelet activation [56]. After a 7-d pretreatment, the combination enhanced eNOS signaling in the aortic wall more effectively than either treatment alone, and showed similar results to aspirin, a standard antiplatelet agent [56].

## 4. Conclusion

In the present study, we report our findings on the antiplatelet effects of a combination extract of sappan wood and red ginger in HFD-induced rats. To our knowledge, this is the first study to demonstrate the antiplatelet effects of the combined extract. Through in vivo experiments, we used an HFD-induced rat model to mimic the risk factors for atherosclerosis-induced CVD pathogenesis in humans. Our focus was on the initial stages of atherosclerosis, which is characterized by elevated levels of cholesterol and triglycerides, as well as platelet hyperreactivity. Therefore, we did not evaluate further stages such as plaque formation in vivo. The antiplatelet effect of the combined extract was demonstrated by a significant decrease in the platelet activation markers P-selectin, RANTES, and PCSK9, as well as the prolongation of bleeding time in HFD-fed rats that received the combined extract. Further studies on the profiling of phytoconstituents in the extracts using LC/MS-MS and network pharmacology are necessary to enhance our understanding of the molecular mechanisms underlying the antiplatelet effects of these combined extracts. Using this approach, Banerjee et al. successfully demonstrated that the combination of these techniques can analyze the lead compounds of the extracts and identify relevant signaling pathways, such as the IRS/Akt/Foxo1 cascade, targeting hyperlipidemia [58]. In addition, after completing the efficacy evaluation, the safety evaluation through toxicity studies are required before progressing to further clinical trials. Notably, acute toxicity studies of sappan wood and red ginger when used individually have been reported; however, an acute toxicity study of their combination has not yet been reported. Nevertheless, our findings suggest the potential antiplatelet effect of the combination extract of sappan wood and red ginger, which could lead to the development of novel antiplatelet agents derived from natural products.

## Figures and Tables

**Table 1:** Variation of extract dose.

Dose variation	Sappan wood extract (mg/200 g bw per day)	Red ginger extract (mg/200 g bw per day)
Dose I	50	200
Dose II	100	400
Dose III	200	800

**Table 2:** Experimental design of the study showing treatment for each group.

Group	Treatment
Control	Normal diet for total 10 weeks and 0.5% CMC 3 mL/200 g bw/day p.o. for 2 weeks
Negative	HFD for 8 weeks and 0, 5% CMC 3 mL/200 g bw/day p.o. for 2 weeks
Positive	HFD for 8 weeks and aspirin suspension 1.5 mg/200 g bw/day p.o. for 2 weeks
Dose 1	HFD for 8 weeks and combination extract dose 1 for 2 weeks
Dose 2	HFD for 8 weeks and combination extract dose 2 for 2 weeks
Dose 3	HFD for 8 weeks and combination extract dose 3 for 2 weeks

**Table 3:** The formulation and calculation of the total caloric content of the HFD used.

Ingredients	HFD (w/v) (%)	Weight of HFD (g)	Calories (kcal)
Goat fat	50	1.31	11.82
Butter	15	0.51	3.74
Cholesterol	2	0.08	N/A
Cholic acid	0.5	0.02	N/A
Fructose	20	1.19	4.17
Coconut oil	12.5	0.42	3.30

*Note:* Weight of HFD (g) = material used × (250 g BW/200 g × 3 mL) × density (g/mL). Caloric content = weight of HFD (grams)/total weight of serving size (grams) × total calories in serving size (kcal).

## Data Availability

The data used to support the findings of this study are available from the corresponding author upon reasonable request.

## Supporting Information

Supporting Figure 1: Certificate of Analysis of *Caesalpinia sappan* L. dry extract.

Supporting Figure 2: Certificate of Analysis of *Zingiber officinale var rubrum* dry extract.

Supporting Figure 3: The effect of administering either CMC (normal and negative), aspirin (positive), or various doses of combined extracts (Dose 1, Dose 2, and Dose 3) on body weight 2 weeks after an 8-week HFD induction period in male Wistar rats. Data are presented as the mean ± SD (*n* = 3 rats/group). ns, not significant denotes statistical significance.
