# Supplementary material for: Antiplatelet Effects of a Combination of Sappan Wood (Caesalpinia sappan L.) and Red Ginger (Zingiber officinale var. Rubrum) Extracts in a High-Fat Diet-Induced Rat Model
Source: Adv Pharmacol Pharm Sci. 2024 Dec 20;2024:5543717. doi: 10.1155/adpp/5543717 (PMC11679274; doi:10.1155/adpp/5543717)
Supplement: Supporting Information — Additional supporting information can be found online in the Supporting Information section. [file 5543717.f1.docx]

Supplementary Figure


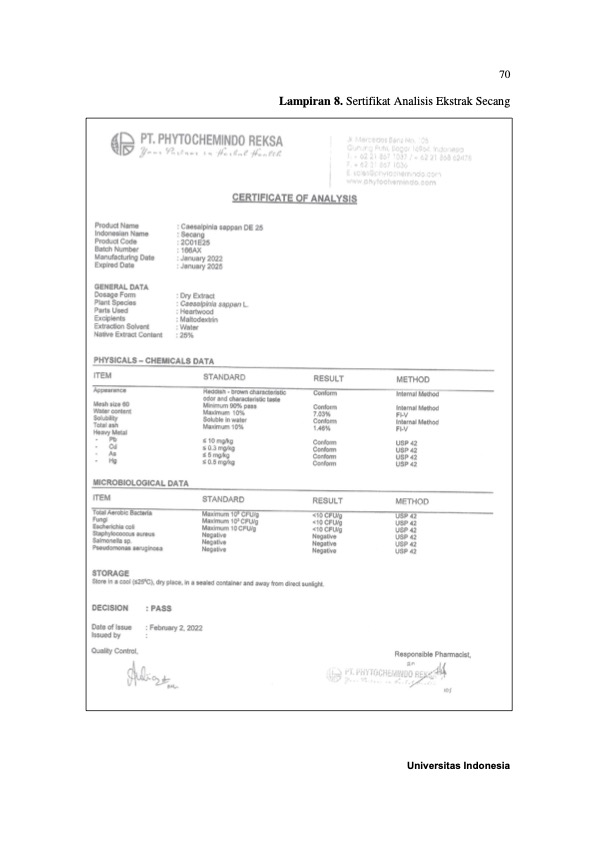


**Supplementary Figure 1.** Certificate of Analysis of Caesalpinia sappan L. dry extract


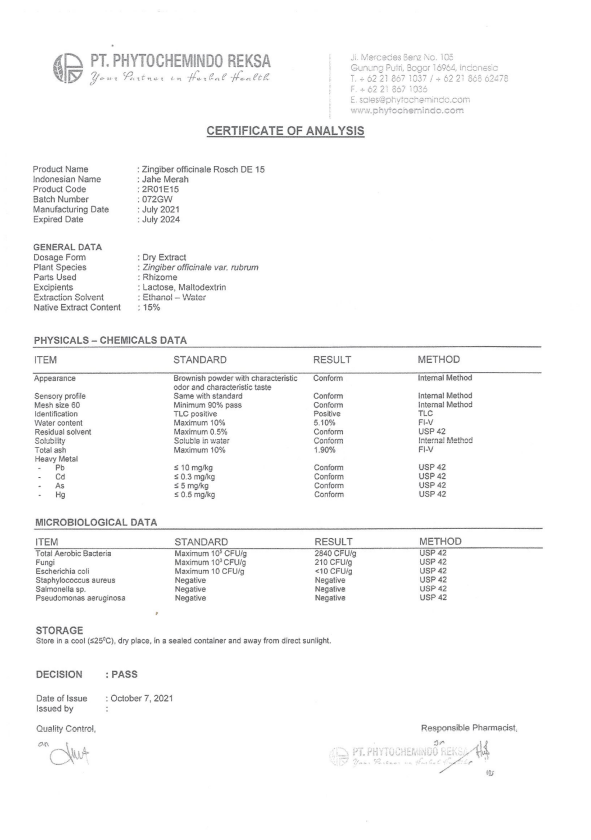


**Supplementary Figure 2.** Certificate of Analysis of Zingiber officinale var rubrum dry extract

**Supplem
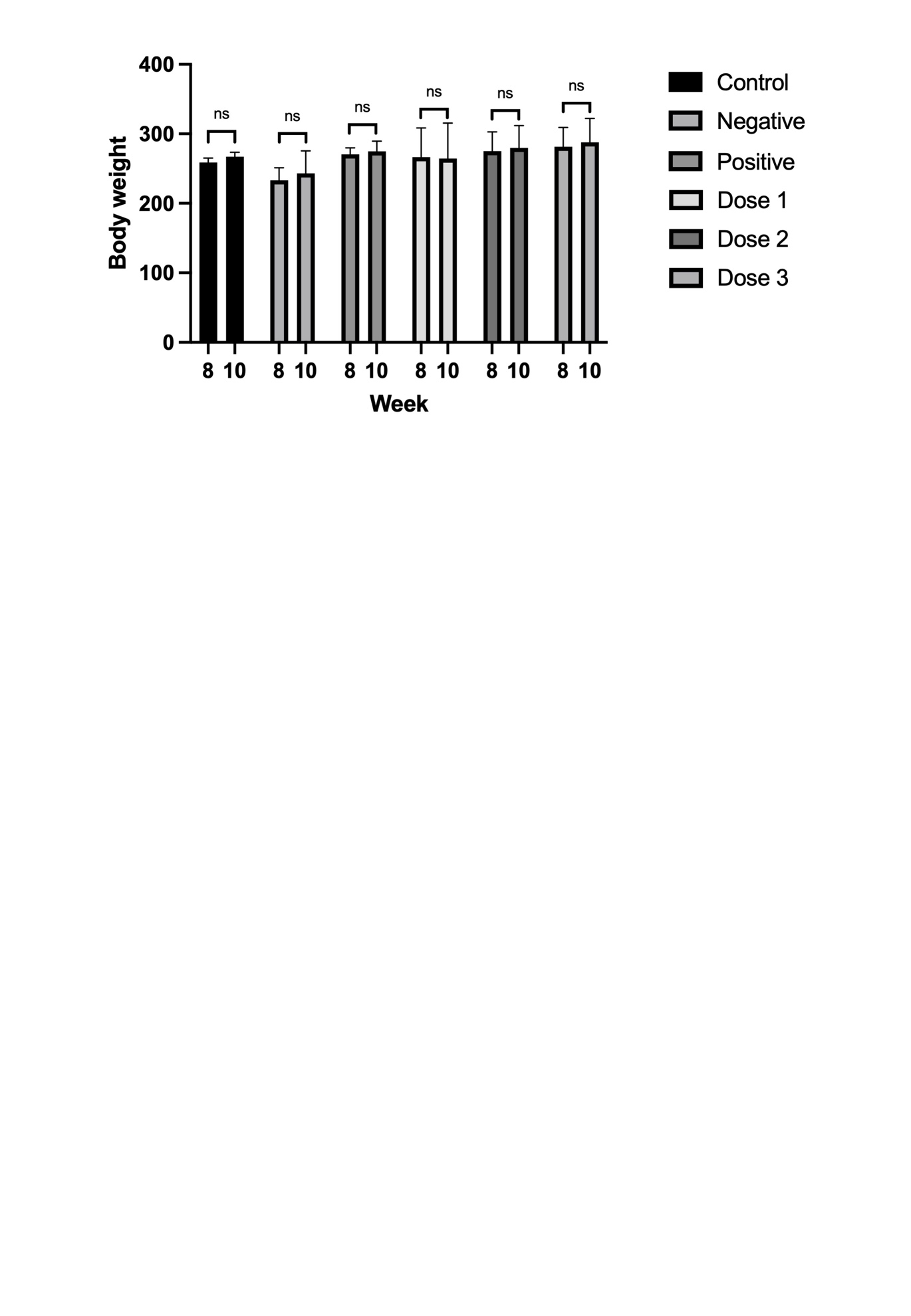
entary Figure 3.** The effect of administering either CMC (normal and negative), aspirin (positive), or various doses of combined extracts (Dose 1, Dose 2, and Dose 3) on body weight two weeks after an eight-week HFD induction period in male Wistar rats. Data are presented as mean ± SD (n = 3 rats/group), ns. not significant denote statistical significance.
